# A database of battery materials auto-generated using ChemDataExtractor

**DOI:** 10.1038/s41597-020-00602-2

**Published:** 2020-08-06

**Authors:** Shu Huang, Jacqueline M. Cole

**Affiliations:** 1 Cavendish Laboratory, University of Cambridge, J. J. Thomson Avenue, Cambridge, CB3 0HE UK; 2 ISIS Neutron and Muon Source, Rutherford Appleton Laboratory, Harwell Science and Innovation Campus, Didcot, Oxfordshire OX11 0QX UK; 3 Department of Chemical Engineering and Biotechnology, University of Cambridge, West Cambridge Site, Philippa Fawcett Drive, Cambridge, CB3 0AS UK

**Keywords:** Energy, Chemical physics, Batteries

## Abstract

A database of battery materials is presented which comprises a total of 292,313 data records, with 214,617 unique chemical-property data relations between 17,354 unique chemicals and up to five material properties: capacity, voltage, conductivity, Coulombic efficiency and energy. 117,403 data are multivariate on a property where it is the dependent variable in part of a data series. The database was auto-generated by mining text from 229,061 academic papers using the chemistry-aware natural language processing toolkit, ChemDataExtractor version 1.5, which was modified for the specific domain of batteries. The collected data can be used as a representative overview of battery material information that is contained within text of scientific papers. Public availability of these data will also enable battery materials design and prediction via data-science methods. To the best of our knowledge, this is the first auto-generated database of battery materials extracted from a relatively large number of scientific papers. We also provide a Graphical User Interface (GUI) to aid the use of this database.

## Abstract (summary)

**Table:** 

Measurement(s)	battery capacity • Voltage • electrical conductivity • Faraday efficiency • energy • Chemical Properties
Technology Type(s)	digital curation • computational modeling technique

Machine-accessible metadata file describing the reported data: 10.6084/m9.figshare.12646277

## Background & Summary

Batteries are essential components of most electrical devices and have accordingly found widespread applications in technological areas such as portable electronics, hybrid electrical vehicles, and stationary storage devices of any size^1^. Given the increasing demand for advanced battery technologies, extensive research is being carried out in this field, especially for the development of advanced materials for safe, efficient, and high-capacity batteries. Over the last few decades, an ever-increasing number of academic papers on battery materials have been published.

These papers are mostly generated from scientists who are reporting their current developments of new materials based on trial-and-error methods. It is accepted that such methods prove frustratingly slow for the discovery of new materials. Finding ways to accelerate the design and development of new materials has thus become an attractive research target. It is anticipated that data science may provide a systematic materials-by-design option that achieves this desired acceleration. In recent years, the development of big-data and machine-learning methods has facilitated huge progress in chemistry and materials science, in fields such as the design and discovery of new catalysts^2^, drugs^3,4^, and photovoltaic materials^5–7^. In 2011, the *Materials Genome Initiative* was launched to deploy big-data methods for the discovery of new materials^8^. This initiative led to the spin-off of many sub-projects, which have shown that data mining can be used to reduce the materials discovery timeline^9–12^.

However, a comprehensive database is essential for the data-driven discovery of new materials. Current data-mining research is mostly based on the datasets that are obtained from high-throughput experiments or theoretical simulations. For theoretical simulations, the *Materials Project* has generated a large computationally derived database of electrode materials for lithium-ion batteries^13^. Many scientists have used this database for tasks such as the prediction of electrical properties for anode^14^ and cathode materials^15–17^. Sendek *et al*.^18^ also used this *Materials Project* database to identify new solid-state electrolytes. Researchers have complemented these theoretical simulation efforts by creating battery databases from high-throughput experiments. For example, NASA has a Prognostics Data Repository which contains three experimental datasets about batteries^19–21^. Severson *et al*. published a battery life cycle dataset, which was then used for predicting battery lifetime^22^. Lao-atiman *et al*. have created a zinc-air battery dataset for use in modelling^23^. The methods used to create these databases were faced with limitations; Severson *et al*. encountered limited sample diversity; Sendek *et al*. were confined to the use of empirical diversity. Another approach is to create a database from scientific literature. Ghadbeigi *et al*.^24,25^ have constructed a battery material database based on experimental data, extracted manually using Datathief (http://datathief.org/). This database was then used by Kauwe *et al*.^26^, who conducted data-driven research using machine-learning tools to predict the capacity of battery materials. However, as their dataset was extracted manually from literature, its size is relatively small. This paper shows how to overcome this problem, by using ChemDataExtractor^27^ to automatically extract data from a huge collection of battery research papers, and thence create a large database of battery materials and their cognate properties.

To the best of our knowledge, this is the first battery materials database that has been auto-generated from data in the literature. We focused on extracting data about battery materials and their functional properties; namely, capacity, conductivity, Coulombic efficiency, energy density, and voltage. ChemDataExtractor version 1.5, which is based on software developments from Cole and co-workers^27,28^, was used for this work, and modified for the specific use of batteries. The workflow for our database auto-generation includes article retrieval, data extraction, data cleaning, data post-processing, and evaluation. The resulting database has potential reuse value for enabling materials discovery in the field of batteries using machine-learning, data-mining and statistical methods.

## Methods

### Article retrieval

Article retrieval is the step required to download academic papers, which is implemented by accessing the Application Programming Interface (API) designed by the publisher for data-mining purposes. The Royal Society of Chemistry (RSC) and Elsevier provide us with access to the full text of their published papers. To download these articles for data-extraction use, the web-scraping package defined in ChemDataExtractor was used, as well as the python HTTP client libraries “urllib3” and “requests”. The working principle of web-scraping is that when visiting a web page, the web browser makes a GET request to ask for the response from the server, so that the server makes decisions on the local user, *e.g*. permitting the paper download. For journal websites, the HTTP request often contains an API key that requires users to sign up to make web scraping a legal and valid process. In addition, the request involves a query search keyword (“battery” in this project) and publication year (1996–2019). Once the request has been granted, the server will send CSS, JavaScript and image format documents to the local clients, as well as the hypertext markup language (HTML) and extensible markup language (XML) files, which contain the structured full content of each article, which is exactly what is needed for data extraction. Accordingly, 197,372 papers were downloaded from the Elsevier Developer Portal (https://dev.elsevier.com/) and 31,689 papers from the RSC (https://www.rsc.org/). As these papers were scraped by simply searching for the word “battery”, all papers that were found to mention the word “battery” or “batteries” in their title, abstract, list of keywords, or the main content, will have been downloaded. However, some of these papers might not be about battery materials; for example, they could be about a battery system that is used in an application, such as robotics, which is irrelevant to our battery materials database. We found that these papers do not generally contain many {chemical, property, value, unit} records for battery properties, normally less than or equal to three records, as one would expect since they are not describing a battery material. This observation enabled a warning flag, “R” (relevance), to be added to the *Warning* field of all data records that are associated with articles in which fewer than three records are extracted. These amount to 11,337 data records (*ca*. 4% of our entire database), which are included in our database by default. However, the user can decide to keep or remove them using the warning flag, “R”, as a filter, should they be wary of this battery relevance issue.

### Document processing

In order to convert the HTML/XML files into plain text, these files were processed using the “reader” package in ChemDataExtractor. These HTML/XML files have hierarchical structures, where the contents exist within different nested tags. For example, the <head> tag contains information such as title, author and DOI. ChemDataExtractor takes advantage of this semantic markup feature to produce plain text according to the title, journal, abstract, keywords, main contents, tables, figures, references, *etc*. As each journal publisher has its specific HTML/XML formatting style to present a scientific paper, a set of rules are specifically defined to process the documents in terms of different journals. By stripping out the embedded markup, the plain text was produced, and a linear stream of elements containing all data in the papers was created. Eventually, these text data were transferred into the Document object that creates sub-objects such as Title, Heading, Paragraph and Citation.

### Natural language processing

Natural language processing (NLP) enables computers to analyse textual data. ChemDataExtractor provides a comprehensive NLP toolkit for the specialised domain of physics, chemistry, and materials science. It exploits state-of-the-art NLP techniques, including tokenisation, word clustering, part-of-speech (POS) tagging and chemical-named entity recognition (CNER). Most of the ChemDataExtractor code remains unchanged for this work, compared with the original version^27^. However, some adaptations are noteworthy since ChemDataExtractor v1.5 was used as the parent tool for this study. In turn, this version was altered to meet the specific inorganic battery materials needs of this project. One such need concerns the fact that many composites and anode/cathode pairs are presented in papers by two chemical compounds, involving symbols such as ‘/’ and ‘−’. Hence, a set of regular expression rules were defined to extract both components of a composite/battery pair. Several new rules were also added to extract more specific chemical names in the domain of battery materials. Also included in the CNER part of ChemDataExtractor are suffixes typical for nanomaterials (*e.g*. “nanoparticles” and “nanocomposites”) as well as suffixes that are common in the battery field (*e.g*. “anodes”, “cathodes”, and “electrolytes”). As battery properties reflect the whole system, including anode, cathode and electrolyte, these suffixes were logged in a data field “Type” for our database, so that they can facilitate the classification of battery materials. The bespoke version of ChemDataExtractor used for this work is available on https://github.com/ShuHuang/batterydatabase/tree/master/chemdataextractor_batteries.

### Relationship extraction

A key step for database auto-generation is the extraction of suitable relationships (*e.g*. relations of chemical name, property name, value and unit) after the document processing and NLP stages. Tools such as ChemicalTagger^29^ attempt to find a universal grammar to interpret all of the scientific information in order to extract relations. Yet, this proves difficult given the large variances of corpora and lexicons. However, with the use of POS taggers and chemical entity recognisers, it is feasible to write specialised regular expression rules for a specific narrow domain such as the field of battery materials. ChemDataExtractor version 1.5, making use of NLP techniques, defines models according to different material properties, by which a chemical record is attributed to a model specifically. One attribute of the model is the property parser, which includes the defined rules for the relationship extraction. To extend data extraction from a single sentence to a broader domain, the interdependency resolution feature of ChemDataExtractor is used for finding the contextual information.

Table 1 shows an example of the battery capacity model object and its class attributes defined in ChemDataExtractor. This model is inherited from a unit model, which is created for the standardisation of the unit format. For a valid model, the value, units, and compound attributes are required to construct the {chemical, property, value, unit} database. As the capacity for a battery is often measured with a certain current and number of cycles, it is also helpful to add their values and units to the dataset. In addition, capacity data that feature information about cycles and current can be useful for predictions such as capacity degradation. In general, capacity also depends on the charging method, such as constant Current, Constant Voltage mode (CCCV), and the cutoff voltages. The method of charging is not included in this database, but it will considered as a part of future work. The parser attribute plays a key role in phrase parsing and data extraction, as it defines the rules to obtain the relationships. For other models, the attributes can also contain solvent, experimental temperature, and apparatus, as the additional reference information for the relevant chemical-value pairs.

**Table 1:** Battery capacity data model and its attributes.

Class Attributes	Data Type	Class Attributes	Data Type
value	string	units	string
specifier	string	compound	model
current_value	string	current_units	string
cycle_value	string	cycle_units	string
parsers	parser lists		

**Fig. 1:**
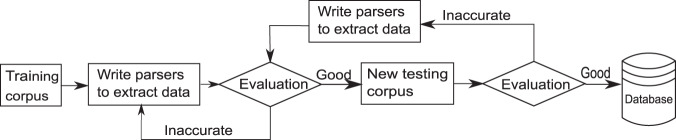
Pipeline used to write a parser.

In this project, we have added five property parsers for data extraction of battery materials (Table 2). These parsers interpret the manually defined grammar into an xpath parse tree from which the data model is constructed. Most of the data models of these properties have only attributes of compound, specifier, value, and units, and parsers compare these with four additional attributes for battery capacity (Table 1). The grammar rule of the parsers was written based on the parser elements shown in Table 3. Using these parser elements, the grammar rule can be combined with the “+” or “|” operators, and the grammar is thus flexible to be updated. Figure 1 illustrates the workflow of writing such a parser. To write a good parser for highly accurate data extraction, each edge case should be considered while a full unit test is performed at the same time. The evaluated results improve with increasingly complicated rules, and certain criteria can be set to determine when the parser is good enough to create a comprehensive database.

**Table 2:** The unit and specifier parse expressions of five property parsers.

Property	Parse Expressions of Units (above) and Specifier (below)
Capacity	W(‘mA’) + W(‘h’) + (W(‘/’) + W(‘g’)) | R(‘^k?g[\—]1$’) | R(‘c?m[\—]3’)
	Optional(I(‘theoretical’) | I(‘specific’)) + (I(‘capacity’) | I(‘capacities’))
Conductivity	R(‘^m?S$’) + R(‘^ c?m[\—]\d+$’)
	Optional(I(‘electronic’) | I(‘electrical’)) + (I(‘conductivity’) | I(‘conductivities’))
Coulombic Efficiency	W(‘%’)
	I(‘coulombic’) + Optional(I(‘efficiency’))
Energy	(W(‘Wh’) + R(r‘^(k?g|m?(L|l))[\—]1$’))
	I(‘energy’) | I(‘energies’)+Optional(’density’)
Voltage	R(‘^m?V$’)+ R(‘^v(s(.)?|ersus)$’) + Optional(R(‘Li|Na|Ag|K’)) + Not(R(‘/s’))
	Optional(I(‘electronic’) | I(‘electrical’)) + (I(‘voltage’) | I(‘potential’))

**Table 3:** The parser elements.

Elements	Description	Elements	Description
R (Regex)	Match text with regular expression	T (Tag)	Match tags
W (Word)	Match case-sensitive token text	I (IWord)	Match case-insensitive token text
Any	Match any single token	H (Hide)	Ignore the matched tokens
Not	Match only if not followed by some text	FollowedBy	Match only if followed by some text
ZeroOrMore	Match zero or more of the expressions	OneOrMore	Match one or more of the expressions
Optional	Match if it exists	SkipTo	Skips to the next occurrence of text

The most important parts of the parser are the chemical identifier, the specifier of a property, its value and units. Table 2 illustrates the units and the specifier parse expression for each property. The capacity units comprise the gravimetric and volumetric units, and specific and theoretical capacities can be distinguished. Even though the Coulombic efficiency has no units, we have added “%” as the unit expression. The energy units comprise the specific energy and the energy density, and the voltage units exclude the case “mV/s”, which is usually used as a scan rate to record spectra. These units will all be eventually normalised to a standard one.

In addition to the differences in unit and specifier parse expressions between each parser, there are also variations in the specific parsing rule. In general, the parsing grammar includes five overall cases: prefix-value-cem, prefix-cem-value, value-prefix-cem, cem-value-prefix, and cem-prefix-value. “Cem” represents the chemical names, “value” contains the value with units, and “prefix” contains the specifier but also text information that might occur near the specifier. By way of example, consider an example sentence: “*The voltage of the lithium battery is 3.4V*”; this can be matched to a prefix-cem-value, where the prefix represents “The voltage of”, the cem represents “lithium”, and ‘3.4V’ is the value.

**Fig. 2:**
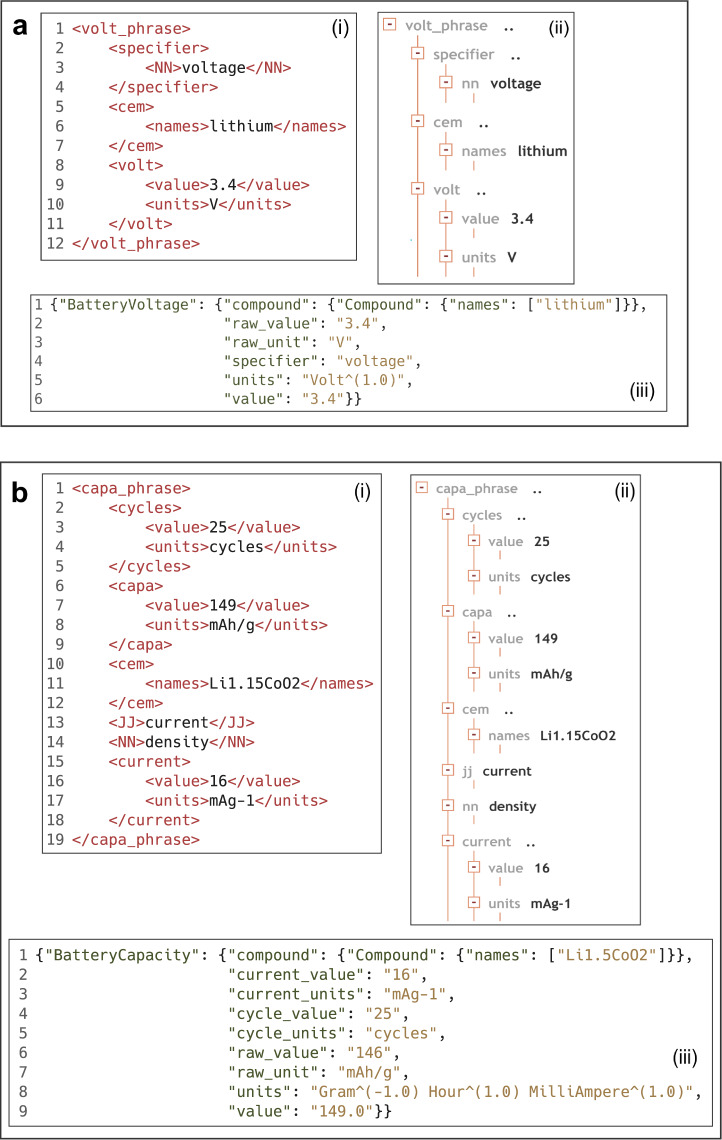
The (i) xpath code, (ii) parse tree, and (iii) the output of the property data of (**a**) battery voltage and (**b**) battery capacity.

Given the same sentence “*The voltage of the lithium battery is 3.4V*”, Fig. 2a shows the XML parse tree (i, ii) and the output of voltage data as a Python dictionary format (iii). The voltage parser interprets the voltage as the specifier tag, and the chemical name “lithium” is within in the tags <names> and <cem>. The value and units are embedded by the tags <value> and <units> within the <volt> tag, while the whole structure is a sub-tree of the element <volt_phrase>. In the ‘BatteryVoltage’ dictionary, the keys contain both the “raw_units” and “raw_value”, and the “unit” and “values”. The “values” and “units” are the post-processed outcomes after the raw value and raw units are standardised in ChemDataExtractor. The capacity property occurs most frequently in this database auto-generation procedure, and it is often measured with a given current density or a certain number of cycles. The battery capacity parser is more complicated than the others. In addition to a more complex parse rule to improve the precision and recall, we defined two extra properties, *i.e*., “current” and “cycle” in the capacity parser, where we used the “SkipTo” function to enable the extraction of these properties followed by a capacity. Figure 2b illustrates how a property model is created given the sentence “*The maximum discharge capacity after 25 cycles is 149 mAh/g for Li*_*1.15*_*CoO*_*2*_*particles at a current density of 16 mA g*^*−1*^*at room temperature*”. In this <capa_phrase> parse tree, the tags <cycles>, <capa> and <current> appear in sequence. The ‘BatteryCapacity’ dictionary also includes the information of current and number of cycles, but the units are not standardised since “cycle(s)” is not a real unit, whereas the current in a battery publication also includes “C-rate”, which cannot be standardised. The C-rate is the current that reflects how fast the battery is charging or discharging. For instance, a “2C” rate means that the current will charge/discharge the entire battery in half hour, while a battery with “C/5” rate charges/discharges in 5 hours.

In summary, the rule-based phrase parsing method is able to extract the {chemical, property, value, unit} relations, and it is also sufficiently flexible to be updated for any specific use. A complex parse rule is likely to achieve both high precision and recall for a battery database. However, the rule-based parser is strict in its requirement to match the correct contents, and it can fail when a minor mismatch occurs. Also, the specifier, compound and values will not occur in a single sentence or paragraph, in some cases, thus decreasing the recall of phrase parsing. To solve this problem, the interdependency resolution part of ChemDataExtractor is introduced. This interdependency resolution feature has two goals. One is the extraction of information from the context rather than a single sentence; the other is the identification of chemical records that are represented as abbreviations or labels, which can lead to disambiguation. To obtain the contextual information, ChemDataExtractor follows the logic that a chronological list of records is stored, after which information is extracted from the heading, previous sentence, product, and title compound record in an attempt to determine the relations. This works well, especially for the synthesis of a compound^27^. Another issue of phrase parsing is that writing a perfect grammar rule is relatively time consuming. Rule-based parsing requires a great deal of effort to improve the performance of data extraction, especially when a huge volume of data are involved.

A semi-supervised probabilistic approach^28^ based on the Snowball algorithm^30^ provides a potential way to address this problem. However, this algorithm processes many individual sentences, while battery materials information tends to span far beyond a single sentence. Moreover, the Snowball algorithm uses bootstrapping and it is therefore a high-precision, low-recall method. The recall of the battery materials database was already posing a challenge given the need to extract five properties as well as the chemical information. Thus, the rule-based parsing approach was used exclusively for this project.

### Data post-processing & augmentation

Raw data records that emanate directly from ChemDataExtractor contain a range of invalid data including incomplete chemical names, inaccurate specifiers, and/or incorrect values and units. Several remedial steps are therefore implemented prior to the final database curation: data cleaning, standardisation of value and units and normalisation of chemical names. The workflow that defines the data cleaning rules was characterised by “testing and updating”. In other words, by manually checking the general tendency of the common incorrect data, the rules were refined and updated. While the data cleaning process can lead to a certain loss of data, it greatly improves the accuracy of the database. For instance, chemical names containing special symbols, or ending with abnormal words such as “mole”, were cleaned from the database. All of these chemical names were then normalised by ChemDataExtractor as well as a materials parser^31^, so that these chemicals can be presented as elements and the number of these elements, which make it easier to process for a future prediction task. The chemical compounds that could not be normalised were all removed. Likewise, property values which were much higher or lower than the average value, or records with a specifier outside of the battery domain, were also removed. For example, we set the limit of capacity value as a maximum of 5000 mAh/g and a minimum of 0 mAh/g, since a value outside of this range is not likely in the area of battery materials. For other properties, the lower and upper bounds of voltages are 0 and 8 V, energy data are between 0 and 5000 Wh/kg, Coulombic efficiency is within 0% to 100%, and conductivity is not greater than 100 S/cm and not smaller than 10–20 S/cm. While the majority of data records with values within these ranges, but near their limits, appear to be correct, manual spot checks on our database showed that they carry more of a risk of being erroneous than data on property values that lie far from such boundaries. This is particularly true for voltage as values near the limit are likely to refer to the cutoff voltages, instead of the average voltage that is associated with the material itself. Given the slightly higher risk of such data being erroneous, a warning flag, “L” (Limit), was assigned to each data record, whose property values lie within a “near limit” region, as defined by the ranges: 0-20 and 3000-5000 mAh/g for capacity, 0–1 and 5–8 V for voltage, 0–100 and 3000–5000 Wh/kg for energy, 0–20% for Coulombic efficiency, and 10–100 S/cm for conductivity. This *Warning* data field allows the user the option to decide to keep or filter out these ‘near limit’ data for their own specific database applications; this carries the understanding that 54,928 data records (*ca*. 19% of our entire database) will be lost by adopting this option, in return for a very modest (<5%) reduction in erroneous data.

A data augmentation process was then performed on the cleaned data, whereby new data were derived from the literature-extracted data using formulae that relate several of the target properties. For example, the specific energy (unit: Wh/g) in the battery domain can be calculated from the voltage (unit: V) multiplied by specific capacity (unit: mAh/g); thus, energy data can also be derived according to this relation. The equation is given by:

1 $$Energy(Wh/g)=Capacity(mAh/g)\ast Voltage(V)/1000$$

This process not only accrues the total amount of information in the auto-generated database, it also levels out a bit the number of data that are acquired for each property which is quite different from each other. For example, most documents have capacity and voltage which can be extracted for the database, but energy or conductivity property specifiers are not so often mentioned in the text of papers. This data augmentation step is implemented at the final stage of post-processing.

A graphical user interface (GUI) was made to help visualise the database and thence aid the reuse of its data. This GUI provides a tabular view of the full database as well as figures for visualisation.

## Data Records

The database can be downloaded from *Figshare*^32^, and it has been presented in three formats: SQL, CSV and JSON. The GUI application integrates the SQLite database in its source code. Table 4 provides an overview of the data records. *Extracted_name* is the normalised compound name as a list of dictionaries, in which each item of the list represents a chemical compound of a composite if it exists, and each dictionary consists of chemical elements and the number of them. *Value* and *Unit* are the values of the chemical property that were normalised through the unit model, and were then converted into a standard unit in the final version. The data in their originally extracted form are listed in records, *Raw_value* and *Raw_unit*, which sometimes contain multiple values. In those cases, each value is distributed to each chemical name one-by-one if there also exist multiple names. If there is only one chemical name, all of the raw values are assigned to this name, and *vice versa*. For properties such as conductivity, the value is usually expressed as a range that depicts a maximum and minimum window. In this case, the max and min values are extracted and stored in two data records respectively. For the many cases where multiple values arise from the same paper, these data need to be distinguished since they generally relate to the presentation of series of data within a given study. Access to series of data on battery materials could be particularly helpful to certain database users. To this end, our database is highly pertinent since 117,403 data records (*i.e*. 40% of our entire database) relate to series of data. Thus, a warning flag, “S” (Series), is provided within the *Warning* data field of our database, so that users can search on the DOIs of these papers and the dependent variable of the data series that interests them. “S” is assigned to the *Warning* data field for each data record where the values appear more than three times with the same chemical name, property and DOI. Two other warning flags may be in this *Warning* data field: warning flag, “R” (Relevance), that cautions the user on the relevance of the 11,337 data records that are more likely to have been extracted from papers on batteries but which are not about battery materials; and warning flag: “L” (Limits), which is assigned to data records containing property values that are near to their minimum or maximum limits. The majority of the 54,928 data records that contain “near limit” values are valid, but their “near limit” property values are more likely to constitute erroneous data compared with values of properties that lie well within their limits. Mixed warnings, such as “LS” and “RL”, are also possible for a given data record. More information about warning flags, “R” and “L”, are provided elsewhere in the paper, in sections *Article Retrieval*, *Data Post-processing & Augmentation* and *Technical Validation*. The data record, *Type*, stores the *ca*. 9,000 data on each material type (*e.g*. anode, cathode, electrolyte) that have been extracted from the literature. The energy data are classified as “CDE” or “Calculated” in *Tag*, according to whether these data were extracted from text using ChemDataExtractor or calculated from capacity and voltage via the data augmentation process. *Specifier* is the property specifier recognised by the parser. The *Info* record contains additional information about a material property record, such as the cycle and current value that is measured together with capacity. In the current version of database, the *Info* value is labelled as ‘*None*’ except where it pertains to battery capacity. For validation use, a *Correctness* data record was incorporated into the database; this indicates whether the extracted data are true or false, a judgement that has been determined manually.

**Table 4:** Summary of data records.

Data	Description	Data type
Property	Material property types	String
Name	Chemical compound names	String
Extracted_name	Normalised chemical name	List of dictionaries
Raw_value	Extracted value from text	String
Raw_unit	Extracted unit from text	String
Value	Normalised value by CDE	Float
Unit	Normalised unit by CDE	String
Tag	Text or calculated data	String
Info	Additional information	List of dictionaries
Type	Type of materials	String
Specifier	Specifier of property	String
DOI	Source article DOI	String
Journal	Published journal	String
Date	Published date	String
Title	Source article title	String
Correctness	Correctness of data	String
Warning	Warning	String

## Technical Validation

The evaluation metrics used in this study are precision, recall and F-score. Precision is the fraction of the correct (“True”) data in the database, recall is the fraction of the data relation that is extracted from the entire records in papers, and F-score is the harmonic mean of precision and recall. The metrics are given by:

2 $$Precision=\frac{TP}{TP+FP}$$

3 $$Recall=\frac{TP}{TP+FN}$$

4 $${\rm{F}} \mbox{-} {\rm{score}}=2\cdot \frac{Precision\cdot Recall}{Precision+Recall}$$

where TP denominates true positive, FP false positive, and FN false negative.

As mentioned previously, a *Correctness* column was added to the database for validation use. By shuffling the database randomly, a total of 500 data records were used as a test dataset for evaluating precision. Since the number of data records for certain property types in this test dataset is much smaller compared to the other properties, more data records with those properties were added manually. For a single data record, it was assigned as “True” if the compound name, value, property and unit were matched to the original text from the paper. The record was classified as “False” if the errors were of one of the four types: “Incomplete composites” (F1), “Incorrect name” (F2), “Incorrect match” (F3), and “Interdependency error” (F4). These manually determined True/False values were added to the *Correctness* column, which can also be found in the GUI by sorting *Correctness*. From these 500 random data records, 51 records with different DOIs were selected for the estimate of recall. For each DOI, the number of relations in the source paper was counted, and then we compared it with the number of data records extracted in our database. Recall is thus determined as the fraction of relations which were extracted from the entire records in original papers. The details of the recall validation results can be found in the Supplementary Information^33–83^.

The precision and recall for the five examined material properties are shown in Table 5. The overall precision is 80.0%, with precision on individual properties ranging from 75.7–83.3%. 80% is generally considered to be comparable to human error for manual data extraction, while the small range quoted (Δ = 7.6%) evidences good consistency across all properties. Conductivity and energy are the two properties with slightly lower precision of around 70%. This is a reflection of the relatively small number of conductivity and energy data, which limits the overall performance of this database. The overall recall (59.1%) means that only about three fifths of the data records were extracted from text; this is due to the somewhat strict criteria applied to the data cleaning process after the data extraction stage.

**Table 5:** Precision, recall, and F-score values of the database for the five material properties.

Properties	Precision	Recall	F-Score
Capacity	83.3%	63.2%	71.9%
Voltage	79.0%	51.9%	62.6%
Conductivity	76.0%	47.1%	58.2%
Coulombic Efficiency	77.6%	63.8%	70.0%
Energy	75.5%	66.7%	70.8%
Overall	80.0%	59.1%	68.0%

Table 6 shows the four types of errors that may lead to a restriction in precision. Most errors arise from an “incorrect match” (F3), which typically occurs in sentences that contain more than one chemical compound or property value, where the parser fails to attribute the data to the correct one. The second most common source of error arises from “incomplete composites” (F1), which means that only a part of the entire composite or device pairs is extracted. For instance, only the name “rGO” is extracted for the composite material, “N-ZnSe@rGO”. The error “incorrect names” (F2) refers to errors such as invalid chemical names which should be removed. Both F1 and F2 are a known problem of CNER. In due course, an improvement in CNER could help improve the accuracy of the database. Interdependency error accounts for 7% of the data extraction errors. This, is expected, owing to the logic of the interdependency rule. However, while the interdependency logic restricts the database precision, it greatly improves its recall, so it should not be abandoned.

**Table 6:** Individual error sources errors of the data and their percentages.

Error sources	Proportion
Incomplete composites (F1)	31%
Incorrect names (F2)	10%
Incorrect match (F3)	52%
Interdependency error (F4)	7%

Nonetheless, the relatively modest overall recall is reasonable, when considering that maintaining a high precision was the priority, *i.e*. ensuring that the information that is entered into the database is indeed correct. The property parser rules are sufficiently specific to each property, such that most of the remaining errors that withhold a higher precision are systematic in their origin. Thus, the database afforded seems to have the best precision that we can obtain without reducing the size of the dataset substantially; if we impose even stricter parser rules, this would naturally increase the precision, but it would lead to a great loss of data.

To this end, our database auto-generation methods have been geared to afford a database that is optimally ‘fit for purpose’ for data-driven materials discovery. For example, a user may wish to employ this database as a source for a data-driven ‘design-to-device’ operational pipeline^11^ where data are progressively filtered down to a small short-list of lead candidates for a target material application. In such a scenario, database entries that are in fact incorrect will likely be filtered out naturally during downstream analysis, while a data source that carries a large number of data is imperative for such a data-driven task. However, it needs to be considered that this database may need to serve a range of purposes depending on the user motivation. For example, a user might want a simple ‘look-up’ database where quality control is imperative but the property sought is common. In such a scenario, precision is valued over number of data. The aforementioned warning flags, L, R and S, account for this diversity in user motivation, by providing an option for users to remove data which might be circumspect owing to their values being: near their extreme limit, questionable in terms of relevance to battery materials, or being part of a data series. While use of these warning flags will remove a lot of data, the database afforded would have a higher overall precision of *ca*. 85%; an increase of 4.2% or 0.8% from use of the “L” or “R” warning flag, respectively.

The default 80% overall precision of our database can be compared holistically to results from NLP-based approaches that auto-generate experimental databases for materials science in other fields, albeit these are currently few. Elton *et al*.^84^ have created a database with chemical-property relationships using word-embedding techniques. This task is slightly different, in that they only capture properties as pre-defined target words (e.g. “non-toxic”) in the database, which has a limited range compared to the “value” and “units” that are identified and enumerated using tools such as ChemDataExtractor^27^. Since Elton *et al*. are dealing with words, they employed a similarity-matching process to validate their data extraction rather than a precision metric. Court and Cole^28^ used an earlier version of ChemDataExtractor (v1.3)^27^, together with the modified Snowball algorithm^28,30^, to create a database of Curie and Néel temperatures for magnetic materials, and achieved a precision of 73%. This precision is slightly lower than that in our database, while our data extraction process is even more complicated, especially with regards to the fact that our database contains five distinct properties compared to the two temperature properties of the magnetic database. The extraction of more properties will inevitably increase the complexity of the sentence parsing that is needed, since researchers tend to use different styles to describe different properties. Note that a precision of less than 80% for a database has been shown to be entirely sufficient for materials discovery using data-driven ‘design-to-device’ operational pipelines^11^. This is because any ‘rogue data’ in the database is mitigated by the nature of the downstream analysis. For example, the aforementioned database of Curie and Néel temperatures with 73% precision has successfully reconstructed phase diagrams of magnetic materials and predicted phase-transition temperatures using machine-learning (ML) methods^85^. ML methods will naturally filter out erroneous data as outliers via the intrinsic nature of their data analytics procedure. Meanwhile, Cooper *et al*.^7^ were able to discover suitable pairs of light-harvesting materials for photovoltaic applications via a data-driven ‘design-to-device’ approach that employed a database of *λ*_*max*_ values which formed part of an NLP-generated database of UV/vis absorption spectral attributes^86^. They used a different type of downstream analysis: one that employs a sequence of encoded forms of structure-property relationships to screen a database for materials, whose property characteristics optimally suit a target application. The sequential procedure successively filters through smaller and smaller sub-sets of the original database that obey each structure-property relationship, until the data sub-set becomes so small that a lead candidate material emerges, bearing all of the structure-property relationships required for the targeted application. The intrinsic nature of this filtering process disregards erroneous data since they do not comply with established structure-property relationships. Thus, the nature of the downstream analysis successfully mitigates the non-perfect precision of a database.

The fully processed database contains a total number of 292,313 records. This comprises a total of 214,617 unique pair-wise data relations; thus 77,696 of the data extracted from the literature have redundancy, which means that these data have the same chemical name and property values, but can be extracted from different papers. Note that there might still be differences not captured by ChemDataExtractor that can cause a variation in property, even though they have the same values. Thus, we provide both a merged and full version of the database, while the unmerged form is the default option. In total, there are 17,354 unique chemically named entities in the database. Table 7 shows how many of the five properties sought have been found and extracted for this total number of compounds, classified into the number of chemicals that have a certain number of properties. While most chemicals have only one or two properties, less than 10% of compounds have more than three. This table also shows the impact of the data augmentation step, from which an increasing number of materials that have more than two properties can be seen. Thus, data augmentation greatly improves the data correlation behaviour in the database. Table 8 illustrates the total number of data records that correspond to each property. Most of these records consist of voltage and capacity, which seems feasible, given that battery scientists focus their research on the improvement of capacity with the measurement of voltage; as such, almost all battery research involves the measurement of voltage-capacity relationships. Conversely, the scarcity of conductivity data is most likely to be intrinsic to the property measurement itself. In this project, conductivity does not distinguish between ionic and electronic conductivities. It is often measured for an electrolyte material, yet rarely tested in the context of anode or cathode materials. However, the anode and cathode are key components of batteries and they are therefore the subject of numerous studies. In comparison, fewer studies focus on electrolytes; this reduces the amount of conductivity data that can be extracted by ChemDataExtractor. The amount of data on Coulombic efficiency is similarly modest in comparison. This stands to reason since this property is usually expressed within the figures of a paper, and this information is often not duplicated in the text, in order to avoid repetition in a paper; in such cases, it is not detected by ChemDataExtractor. As mentioned earlier, the energy data have been augmented via the derivation of data from the availability of the extracted voltage and capacity data and its inherent relationship to energy. Conversely, the number of energy records is larger than those of conductivity and Coulombic efficiency. In summary, the database contains a relatively large number of chemical compounds, while the difference in the number of chemical names per property is also large.

**Table 7:** Number of chemicals for which data on one to five properties have been acquired using ChemDataExtractor (CDE) or were derived from CDE-extracted data (CDE + calculated data, CDE data only).

Number of properties	Number of chemical compound (CDE only)	Number of chemical compound (CDE + Calculated)
1	11,242	11,242
2	3,929	1,351
3	1,562	3,024
4	414	1,403
5	207	334
Total	17,354	17,354

**Table 8:** Number of data records for each property.

Property	Total number of data records
Capacity	144,359
Conductivity	7,168
Coulombic Efficiency	11,003
Energy	15,543
Voltage	114,240
Total	292,313

**Fig. 3:**
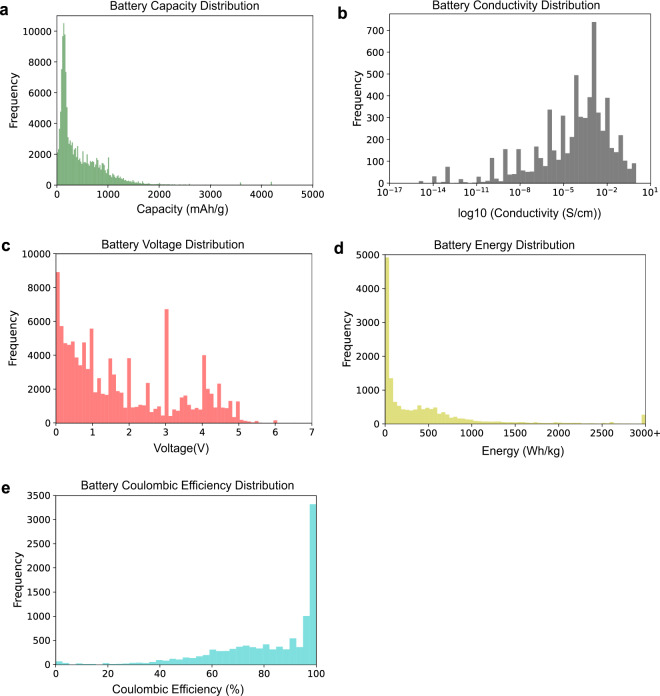
The data distribution of the five properties for battery materials examined in this study: (**a**) capacity, (**b**) conductivity, (**c**) voltage, (**d**) energy, and **(e**) Coulombic efficiency.

**Fig. 4:**
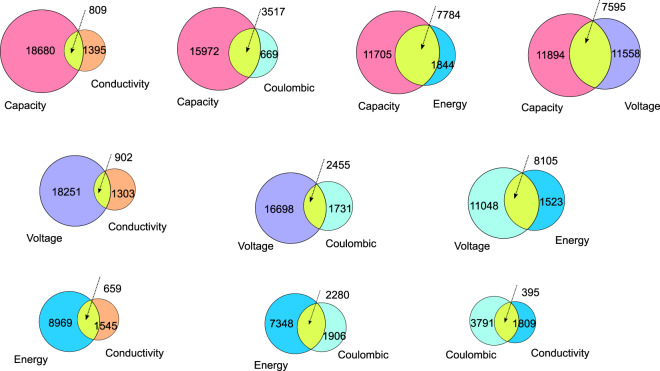
Venn diagrams of the data correlation between all possible pair-wise permutations between the five examined materials properties.

Figures 3 and 4 illustrate an overview of these database proportions. Figure 3 presents the histograms of the data distribution for the five examined battery material properties (capacity, conductivity, voltage, energy, and Coulombic efficiency). Figure 4 shows Venn diagrams that describe how many chemicals share two properties; this provides a guide as to the extent of data correlation.

## Usage Notes

The database has been presented in both relational and non-relational formats including SQL, CSV and JSON. There are merged and full versions of the database in these formats; the full version is the default option. They can be easily queried by database languages, SQL or Mongo, as well as a programming language such as Python, R, Java, or Matlab. The structured features of the data model make it easy for scientists to add more material-property relationships to the data, as well as to perform queries on database (*e.g*. read, search, update, and delete). The database can be found in *Figshare*^32^. The most intuitive way to view and reuse the data is by using the GUI that we have provided in this work. It contains a *Table* section in which data records can be presented according to any data type and any sorting. Users can directly type in material name, property, or DOI in the search box of the GUI to look for a target material and property. One can also search exclusively for data that carry one or more of the warning flags, S, L and R. Users can view a basic statistical analysis of the whole or part of the database via the *Figure* GUI, which includes pie chart, bar chart, histogram, and Venn diagram display options. The installer of the GUI application can be downloaded from *Figshare*^32^. Users can add more data as more papers are published, by following the data extraction pipeline (https://github.com/ShuHuang/batterydatabase); this pipeline can also be used as a guideline for the data extraction of other material properties.

## Supplementary information

Supplementary Information

## Data Availability

The source code used to generate the database is available at https://github.com/ShuHuang/batterydatabase. The code of ChemDataExtractor 1.5 that has been modified for database auto-generation in the battery domain is available at https://github.com/ShuHuang/batterydatabase/tree/master/chemdataextractor_batteries. The GUI application source code can be found at https://github.com/ShuHuang/batterygui.
